# Noise exposure while commuting in Toronto - a study of personal and public transportation in Toronto

**DOI:** 10.1186/s40463-017-0239-6

**Published:** 2017-11-23

**Authors:** Christopher M.K.L. Yao, Andrew K. Ma, Sharon L. Cushing, Vincent Y.W. Lin

**Affiliations:** 1 Department of Otolaryngology-Head and Neck Surgery, University of Toronto, Toronto, Canada; 2 Department of Otolaryngology-Head and Neck Surgery, Hospital for Sick Children, Toronto, Canada; 3 Department of Otolaryngology-Head and Neck Surgery, Sunnybrook Health Sciences Centre, Toronto, Canada; 4 Sunnybrook Research Institute, Sunnybrook Health Sciences Centre, 2075 Bayview Ave., Room M1 102, Toronto, ON M4N 3M5 Canada

**Keywords:** Noise-induced hearing loss, Noise dosimetry measurements, Mass transit, Commuting

## Abstract

**Background:**

With an increasing proportion of the population living in cities, mass transportation has been rapidly expanding to facilitate the demand, yet there is a concern that mass transit has the potential to result in excessive exposure to noise, and subsequently noise-induced hearing loss.

**Methods:**

Noise dosimetry was used to measure time-integrated noise levels in a representative sample of the Toronto Mass Transit system (subway, streetcar, and buses) both aboard moving transit vehicles and on boarding platforms from April – August 2016. 210 measurements were conducted with multiple measurements approximating 2 min on platforms, 4 min within a vehicle in motion, and 10 min while in a car, on a bike or on foot. Descriptive statistics for each type of transportation, and measurement location (platform vs. vehicle) was computed, with measurement locations compared using 1-way analysis of variance.

**Results:**

On average, there are 1.69 million riders per day, who are serviced by 69 subway stations, and 154 streetcar or subway routes. Average noise level was greater in the subway and bus than in the streetcar (79.8 +/− 4.0 dBA, 78.1 +/− 4.9 dBA, vs 71.5 +/−1.8 dBA, *p* < 0.0001). Furthermore, average noise measured on subway platforms were higher than within vehicles (80.9 +/− 3.9 dBA vs 76.8 +/− 2.6 dBA, *p* < 0.0001). Peak noise exposures on subway, bus and streetcar routes had an average of 109.8 +/− 4.9 dBA and range of 90.4–123.4 dBA, 112.3 +/− 6.0 dBA and 89.4–128.1 dBA, and 108.6 +/− 8.1 dBA and 103.5–125.2 dBA respectively. Peak noise exposures exceeded 115 dBA on 19.9%, 85.0%, and 20.0% of measurements in the subway, bus and streetcar respectively.

**Conclusions:**

Although the mean average noise levels on the Toronto transit system are within the recommended level of safe noise exposure, cumulative intermittent bursts of impulse noise (peak noise exposures) particularly on bus routes have the potential to place individuals at risk for noise induced hearing loss.

**Electronic supplementary material:**

The online version of this article (10.1186/s40463-017-0239-6) contains supplementary material, which is available to authorized users.

## Background

Hearing loss (HL) is one of the 3rd most prevalent health problems in the world, with the World Health Organization (WHO) estimating over 360 million people living with disabling HL, and over 1 billion young individuals (age 12–35) at risk of hearing loss due to recreational exposure to loud sounds [1]. In the United States, estimates of the prevalence of HL have ranged from 0.5–26% [2–4], garnering the attention of the Center for Disease Control and Prevention (CDC) in targeting reduction of hearing loss as a key focus in their Health People 2020 health initiative [5].

Furthermore, we have only recently begun to fully understand the impact of hearing loss, with studies demonstrating a decline in employment and productivity [6, 7], stress [8], annoyance, sleep deprivation, and disturbance of psychosocial well-being [9, 10]. It has been estimated the total lost in productivity from HL approximates $615 billion US dollars and that a reduction in 20% of hearing loss may result in an economic benefit of $123 billion from loss productivity in the United States [11].

Of individuals with disabling HL worldwide, approximately 16% is attributed to noise-induced hearing loss (NIHL) [12]. NIHL is well known to be caused by chronic exposure to excessive noise, making it potentially preventable. After all, noise exposure is a product of the sound pressure level weighted according to the sensitivity of human ears to different frequencies (A-weighted decibels (dBA)) and time exposure. Several organizations have set out to prevent noise-induced hearing loss, by establishing recommended noise exposure limits (Table 1) [13–16]. Models based on these recommendations were then derived to predict the amount of NIHL based on specific noise-exposure levels [17]. The US Occupational Safety and health Administration (OSHA) exposure limit represents a regulatory standard, permitting an exposure of 85 dBA for 16 h a day, however its standards are known not to protect every worker from suffering (NIHL). Instead, more conservative models were developed including the US National Institute for Occupational Safety and Health (NIOSH) and the US Environmental Protection Agency (EPA) limits, which aim to protect 92–98% of the population from NIHL. Their main difference lies in that NIOSH limits were developed to protect against occupational noise exposure over an 8-h workday, whereas the EPA limits set to prevent NIHL from everyday noise over 24 h. The EPA suggests that chronic exposure of 80.3 dBA for more than 160 min per day was likely to produce hearing loss in exposed individuals. Although this offers a guideline, it only accounts for chronic noise exposure at a static intensity, and does not capture the potential traumatic effects of impulse noise exposure [18].

**Table 1:** Recommended noise exposure thresholds

Duration	WHO /EPA (dB)	OSHA (dB)	NIOSH 1997 (dB)
8 h	75	90	85
4 h	78	95	88
2 h	81	100	91
1 h	84	105	94
30 min	87	110	97
15 min	90	115	100
7 min 30 s	93		103
3 min 45 s	96		106
1 min 53 s	99		109
56 s	102		112
28 s	105		115
14 s	108		118
7 s	111		121
4 s	114		124

Recently, excess noise has been highlighted as a major environmental exposure in urban areas [19]. Above and beyond NIHL, chronic noise exposure has been associated with hypertension, myocardial infarction, stroke, adverse sleep patterns, and even adverse mental health [20–24]. With more than half of the world’s population now living in cities [25], it is important to characterize contributors of excess noise exposure. One major source of excess noise in urban environments is mass transit. In New York City, a study on their mass transit system noted the loudest exposure to be on the subway, with average time-weighted noise levels averaging 80–90 A-weighted decibels (dBA), and reaching peaks of 106 dBA [26]. Several studies have assessed noise exposure in other mass transit systems, however, few have implemented noise dosimeters, which allow for the calculation of time-weighted sound level averages [27–29].

In this study, we capture the noise exposure experienced by Toronto commuters, including subway, streetcar, buses, cycling and walking in and around Toronto. The Toronto subway system is Canada’s oldest subway system, built in 1954 and the fourth largest in North America with an annual ridership of 538 million [30].

## Methods

Noise levels were measured in the Toronto city area during April to August 2016 on various methods of mass transit including subways, buses, streetcars, private vehicle, cycling and walking. Measurements were carried out with a type II noise dosimeter, (SL355; Extech Instruments, Nashua, NH). Both continuous frequency-weight averages (L_eq_), representing the average noise exposure level over a period of time, and maximum peak noise exposures (L_max_) were captured.

The dosimeter was configured to the OSHA and ISO standards, and calibration confirmed in a sound booth with a sound level calibrator. The dosimeter captures A-weighted sound levels between 60 and 130 dB with peaks up to 93-133 dB. For L_eq_ measurements, sound pressure levels were captured every second. Research staff (CY, AM) carried the dosimeter mic on a collared shirt 2 in. away from the researcher’s ear to provide a representative estimate of personal noise exposure.

### Data collection

All measurements were carried out on weekdays between 7:00 am to 7:00 pm in vehicles as well as boarding platforms of subways, streetcars, and buses. Platform measurements had a target length of 2 min, around the time of vehicles arriving or departing the station. Onboard measurements were carried out over a length of 4 min, where researchers sat approximately in the middle of each transit vehicle. To ensure consistency, measurements on platforms were taken roughly 8–12 in. away from the platform edge near the middle of the platform.

For subway measurements, we accounted for variations in acoustics, station ridership, ambient noise levels, above or below ground stations by collecting in-vehicle measurements along the entire subway path, and collecting 2 platform measurements for each of 55 stations. This covers the busiest platforms along the Bloor line, Yonge-University Line, Sheppard Extension and Scarborough light rail extension. We also collected measurements within 5 streetcar rides, and 2 streetcar platforms along routes throughout downtown and midtown Toronto. Recordings of various midtown bus routes including 10 bus rides, and 13 bus platforms measurements were carried out. We included 5 measurements within a personal vehicle (2009 Honda Civic), along typical commuting routes such as the Don Valley Parkway, and Highway 401 with the windows rolled up and radio background noise turned off. Finally, 7 measurements while cycling and 7 while walking were performed along downtown city core routes.

During the measurements, the type of transit vehicle, boarding area, location of route, and surrounding environments (aboveground or underground) as well as the duration of measurement was captured. Any unusual circumstances during the measurement such as the presence of buskers or construction was noted. The data was then captured onto an Excel file (Microsoft Corp, Redmond, WA), and imported to SPSS Statistics (IBM Corp, Armonk, NY) for data analyses.

### Analyses

We conducted analyses by transit method, compared and computed descriptive statistics for each system by measurement location (in-vehicle vs. platform), and station location (above vs. below ground). We used 1-way analysis of variance (ANOVA) to compare statistical differences in Leq level by transit measurement location, and for subway noise exposures, by subway line and station location. A post-hoc Tukey Honestly Significant Difference (HSD) test was used to determine which means were different. We considered statistical tests significant for values below 0.05.

## Results

Overall, 210 measurements of noise exposure were conducted. Tables 2 and 3 provide the number of measurements, and average time-weighted (L_eq_) and peak (L_max_) sound levels measured at each commuting modality respectively. When time weighted averages are compared, noise exposure was louder on combined measurements of subway and buses than streetcars (79.8 +/− 4.0 dBA, 78.1 +/− 4.9 dBA vs 71.5 +/− 1.8 dBA, *p* < 0.0001). The time-weighted average noise exposure was lower for driving a personal vehicle (67.6 +/− 4.0 dBA) when compared with biking (81.8 +/− 3.4 dBA, *p* < 0.0001) and walking (73.9 +/− 5.4 dBA, *p* = 0.05). Biking also exposed participants to louder time-weighted average noise exposure than walking (*p* = 0.007).

**Table 2:** Average (L_eq_) Noise levels in dBa, by transit type and measurement location: Greater Toronto Area, April–August 2016

	Combined L_eq_ Levels			L_eq_ levels inside vehicle			L_eq_ levels On Platforms			*p*-value*
	No.	L_eq_ +/− SD (dBa)	Range (dBa)	No.	L_eq_ +/− SD (dBa)	Range (dBa)	No.	L_eq_ +/− SD (dBa)	Range (dBa)	
Subway	156	79.8 +/− 4.0	71.1–87.6	45	76.8 +/−2.6	73–84.4	111	80.9 +/− 3.9	71.1–87.6	<0.0001
Streetcar	10	71.5 +/− 1.8	68.5–73.9	8	71.1 +/− 1.9	68.5–73.9	2	72.9 +/− 0.2	72.8–73.1	0.23
Bus	25	78.1 +/− 4.9	68.2–87.6	12	76.3 +/− 2.3	68.2–87.6-	13	79.7 +/− 6.1	73.1–79.5	0.06
Personal Car	5	67.6 +/− 4.0	61.3–70.9							
Bike	7	81.8 +/− 3.4	76.7–88.2							
Walking	7	73.9 +/− 5.4	67.3–80.1							

*the one-way analysis of variance by measurement location

**Table 3:** Peak (L_max_) Noise levels in dBa, by transit type and measurement location: Greater Toronto Area, Apr – Aug, 2016

	Combined L_max_ Levels			L_max_ levels inside vehicle			L_max_ levels On Platforms			*p*-value*
	No.	L_max_ +/− SD (dBa)	Range (dBa)	No.	L_max_ +/− SD (dBa)	Range (dBa)	No.	L_max_ +/− SD (dBa)	Range (dBa)	
Subway	156	109.8 +/− 4.9	90.4–123.4	45	113.3 +/− 2.9	106.2–120.3	111	108.6 +/− 5.3	90.4–123.4	<0.0001
Streetcar	10	108.6 +/− 8.1	103.5–125.2	8	109.9 +/− 8.4	103.5–125.2	2	103.5 +/− 0	103.5	0.33
Bus	25	111.7 +/− 10.3	89.4–128.1	12	103.6 +/− 7.0	89.4–114.4	13	120.4 +/− 5.0	109.1–128.1	<0.0001
Personal Car	5	114.9 +/− 5.5	109.6–122.2							
Bike	7	123.8 +/− 5.5	118.6–135							
Walking	7	111.4 +/− 6.1	103.5–120.2							

*the one-way analysis of variance of Lmax inside vehicle compared with platform

Time-weighted averages on subway platforms were louder than in-vehicle measurements (80.9 +/− 3.9 dBA vs. 76.8 +/− 2.6 dBA, *p* < 0.0001). This difference was not found on buses or streetcars (79.9 +/ 6.1 dBA vs. 76.3 +/− 2.3 dBA, *p* = 0.08; 72.9 +/− 0.2 dBA vs. 71.1 +/− 1.9, *p* = 0.23). Average time spent commuting based on mode of transportation was obtained from the 2011 Stats Canada National Household Survey (Table 4) [31]. Based on this, average commute duration using public transportation was 47 min and 30 s, correlating with an EPA recommended noise exposure of approximately 85 dBA. This level of noise exposure was exceeded in 9% of subway measurements, 12% of bus measurements, and 14% of biking measurements. None of the streetcar, personal car, or walking measurements exceeded this threshold.

**Table 4:** Average commuting times in Toronto (2011 National Household Survey)

	Mode of Transportation	Average Time
TTC	23.3%	47 m, 30s
Car	70.9%	29 m, 18 s
Biking	1.2%	22 m, 48 s
Walking	4.6%	14 m, 48 s

**Fig. 1:**
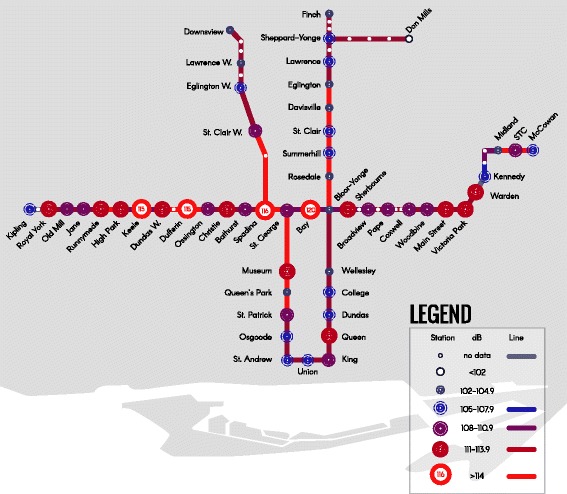
Toronto Transit System Noise Measurements

Peak noise measurements were captured on majority of subway platforms (Fig. 1). Peak noise measurements did not significantly differ between combined subway, streetcar or buses (data not shown). However, the mean peak noise levels were louder in subway vehicles than subway platforms (113.3 +/− 2.9 dBA vs. 108.6 +/− 5.3 dBA, *p* < 0.0001). Whereas, mean peak noise was louder on bus platforms than within buses (120.4 +/− 5.0 dBA vs. 103.6 +/− 7.0 dBA, p < 0.0001). Bus platforms were also found to be on average louder than subway platforms and streetcar platforms (*p* < 0.0001). When personal transport was measured, bikers were exposed to louder peak noise than pedestrians and drivers (123.8 +/− 5.5 dBA vs. 111.4 +/− 6.1 dBA, *p* = 0.02; vs. 114.9 +/− 5.5, *p* = 0.03). For public transport users, the loudest sound measurement came from a bus stop (128.1 dBA), whereas for personal transport users, the loudest peak sound measurement was while biking (135 dBA).

Referring to the EPA noise level thresholds, exposure to peak noise level of 114 dBA for longer than 4 s, 117 dBA for longer than 2 s or 120 dBA for longer than 1 s may place an individual at risk of NIHL. 19.9% of peak subway measurements were greater than 114 dBA, with at least 2 measurements greater than 120 dBA. 20% of peak streetcar measurements were greater than 120 dBA, and occurred during vehicular rides. 85% of peak bus platform measurements were greater than 114 dBA, with 54% being greater than 120 dBA. None of the in vehicular peak bus measurements exceeded 114 dBA. All peak biking noise exposures exceeded 117 dBA, with 85% being greater than 120 dBA. Individuals walking and driving a car were also exposed to peak noise levels greater than 117 dBA (40% in cars, and 14% walking).

Subway platforms were studied in more detail, with comparison between platform noise measurements made between subway lines, platform locations, platform designs, and year built (Table 5). Non-aggregated data can be found as a supplementary table (Additional file 1: Table S1). Line 2 platforms had louder peak noise exposures than Line 1 platforms (111.3 +/− 2.9 dBA vs. 106.5 +/− 3.0 dBA, *p* < 0.0001). There were no other significant differences between subway lines, platform location, or platform design. Mean peak noise levels were louder for subway platforms built between 1960 and 1969.

**Table 5:** Comparison of Subway platform noise exposures by Line, Station Design, Year Built

	L_eq_ on platforms (DBA)	L_max_ on platforms (DBA)	L_eq_ levels in vehicle (DBA)	L_max_ levels in vehicle (DBA)
Subway Line (number of stations)				
Line 1 (26/32)	80.1 +/− 3.5	106.5 +/− 3.0	76.2 +/− 1.9	114.5 +/− 2.5
Line 2 (22/28)	82.8 +/− 2.9	111.3 +/− 2.9	75.6 +/− 1.2	112.1 +/− 2.3
Line 3 (3/5)	76.7 +/− 3.6	106.6 +/− 2.6	83.2 +/− 0.8	110.5 +/− 3.4
Line 4 (2/5)	79.9 +/− 4.3	105.0 +/− 1.5	81.2 +/− 1.0	112.1 +/− 0.7
Platform Location				
Underground (41/53)	81.2 +/− 3.7	108.6 +/− 4.3		
Surface (8/13)	81.2 +/− 3.9	107.8 +/− 4.4		
Elevated (3/3)	76.7 +/− 3.6	106.6 +/− 2.6		
Platform Design				
Central (17/26)	80.0 +/− 3.7	107.0 +/− 5.5		
Side (35/43)	81.4 +/− 3.6	109.0 +/− 3.3		
Year Built				
1990–2009 (2/5)	78.1 +/− 2.6	102.9 +/− 0.6		
1980–90 (4/7)	76.4 +/− 3.1	106.4 +/− 2.2		
1970–79 (6/10)	82.3 +/− 4.0	106.5 +/− 2.5		
1960–69 (27/33)	82.8 +/− 2.6	111.0 +/− 2.9		
1950–59 (13/13)	78.4 +/− 2.8	105.5 +/− 2.5		
1943 (1/2)	84.5	105		

Line 1 = Yonge – University subway line, Line 2 = Bloor – Danforth subway line, Line 3 = Scarborough extension, Line 4 = Sheppard extension

## Discussion

Our findings from this study confer our hypothesis that given sufficient exposure public transportation in Toronto may pose a risk for noise-induced hearing loss. Both the bus and subway had louder mean L_eq_ noise levels (79.8 +/− 4.0 dBA, 78.1 +/− 4.9 dBA) than streetcars, with subway platforms being significantly louder than in-vehicle subway noise (80.9 +/− 3.9 dBA vs 76.8 +/− 2.6 dBA). Furthermore, if we extrapolate the EPA recommended noise thresholds for an average Toronto commuter using public transportation (47 min), we would find that 9% of subway noise exposure and 12% of bus noise exposure exceeded the recommended 85 dBA threshold.

Our most important finding however may be the frequency of which peak noise levels measured in the public transport system exceeded recommended thresholds. Up to 20% of subway measurements had mean peak noises greater than 114 dBA, and up to 85% of bus platform measurements exceeded that threshold, with 54% greater than 120 dBA. Referring back to the EPA noise threshold guidelines, an exposure longer than four second for a 114 dBA noise exposure, and one second of 120 dBA may place the individual at greater risk of NIHL. Peak noise levels were louder in subway vehicle than platforms (Table 3), however, the loudest mean peak (L_max_) noise was found on the bus stop (120.4 +/− 5.0 dBA). Even if this exposure is measured in seconds, it is well known that impulse noise exposure and repeated trauma from noise exposures at this level may place an individual at greater risk of NIHL [32–34]. In fact, animal models suggest that impulse noise exposure may cause hair cell loss more rapidly, and greater hearing threshold shifts than continuous noise exposure [33, 34].

There have only been a few studies looking at dosimetry measurements of noise exposure from public transportation. Neitzel et al. 2009 similarly found that roughly 20% of their subway L_eq_ measurements exceeded the threshold of 85 dBA, however, their mean L_max_ noise measurements ranged from 88.0–90.5 dBA, with their loudest capture noise exposure being 102.1 dBA [26]. This is several orders lower than the L_max_ captured in our study of 128.1 dBA on a bus stop and 123.4 dBA on a subway platform (Table 3). Our measurements were closer to the measurements found on the Bay Area Rapid Transit system in the San Francisco area, with a mean L_eq_ of 82 dBA, 22% of measurements exceeding the threshold of 85 dBA and majority of routes with over half their measurements with L_max_ louder than 90 dBA [27]. Measurements performed in Chicago, also demonstrated routes along the subway system where the noise exposure exceeded the 85 dBA threshold, attributing it to the effects of being in an underground tunnel [28]. In all these transport systems, there is sufficient noise exposure to increase the riders’ risk to NIHL.

Indeed, to adapt and potentially mitigate the level of noise exposure from public transportation, the contributors to loud noise exposure deserve particular attention. Dinno et al. 2011 used a clustered regression analysis to identify train-specific conditions (velocity and flooring), and rail conditions (velocity and tunnels) that may contribute to levels of noise exposures [27]. They found L_eq_ measurements to increase linearly with average velocity by 0.52 dBA/km/h, with the effect tapering to a linear increase of 0.05 dBA/km/h above 53 km/h. Trains traveling through tunnels also increased the L_eq_ by 5.1 dBA, with the type of flooring contributing a small effect to overall mean noise measurements.

Shah et al. 2016 studied the design of New York City subway platforms, finding that overall, curved stations trended louder than straight stations, with L_eq_ noise levels reaching significantly louder intensities at the inbound end of the platform than outbound (89.7 dBA vs 78.7 dBA) [35]. In our study, we found that stations built in the 1960–69 s, when majority of the Line 2 stations were built had louder peak noise levels, whereas the platform design, and location did not play a significant role. It is not known at this time why that decade resulted in subway designs with more intense peak noise exposure, as even older stations did not result in this finding. In addition to the overall layout of the station, there are engineering characteristics such as track curvature, train and rail age, use of vibration reduction methods, as well as environmental factors such as wall material and station size that can contribute to noise exposure while on a subway platform. Specific to train induced noise exposures, engineering studies have described three broad categories of noise: rolling noise, representing the vibration between wheel and rail surfaces; impact noise, representing any discontinuity between the wheel or rail surface; and wheel squeal, representing the friction between wheels sliding against sharp turns [35, 36]. As it may be difficult to address some of the noise derived from existing train paths (curved paths), other endeavours such as the implementation of rail friction modifiers, dampers, and sound barriers may be a more feasible solution [37, 38].

Although most studies have focused their attention on subway transportation, we characterized the noise exposure while using other modes of public transportation including buses and streetcars. To our surprise, although in-vehicle bus measurements mean L_eq_ noise levels were comparable to those previously reported in the New York mass transit system (78.1 +/− 4.9 dBA vs. 75.7 +/− 3.0 dBA), peak L_max_ noise exposure were significantly more intense (120.4 +/− 5.0 dBA vs. 87.8 +/− 7.1 dBA). [26] Certainly, factors such as the distance between the bus stop and the bus play a role, however, with over 85% of bus stop noise level measurements exceeding threshold, more studies assessing the engineering characteristics are required. Recently, the importance of noise exposure within buses has been highlighted by a study demonstrating higher rates of hearing impairment and high blood pressure amongst bus drivers [39].

One of the strengths of this study, was the broad scope of commuting modalities studied. Noise exposure while driving with speeds up to 100 km/h had a L_eq_ of 67.6 +/− 4.0 dBA with peak noise ranging from 109.6–122.2 dBA. Although no prior studies have reported measurements of in-vehicle noise while driving a closed automobile, a study comparing the difference in noise exposure of a top-open and top-closed convertible automobile also depicted the potential for excessive noise above a certain speed [40]. Interestingly, when personal commuting was measured, biking exposed riders to a louder mean Leq noise level than walking or driving (81.8 +/− 3.4 dBA vs. 73.9 +/− 5.4 dBA, vs. 67.6 +/− 4.0 dBA). This also held true for mean peak noise exposures (Table 3). Although the sample size of this was low, and focused around the downtown core, a study mapping out the noise exposure of over 85 bicycling trips in Montreal supported our finding of the potential for significant noise exposure during morning peak traffic hours as well [41]. In general, cyclist have shorter commute times than those using public transit or personal vehicles (Table 4), however, their exposure to louder peak noise also suggest they may benefit from hearing protection. Complicating this decision lies in the fact that hearing is essential for cycling road safety. Other strategies such as developing dedicated bike lanes in low-traffic areas should thus be considered.

Our findings add to the body of literature demonstrating potential sources of noise exposure while commuting. Criticism of these studies have revolved around the cross-sectional design which preclude causality. One study that has attempted to address this gap administered an extensive self-administered questionnaire to over 756 study participants in New York City, finding that at least approximately 32% of participants frequently experienced symptoms suggestive of a temporary threshold shift after using the mass transit system [42]. They also found that two-thirds of their participants reported the use of MP3 players or stereos with an average use of 3.1 h, and that only 14% of participants wore hearing protection at least some of the time while using the mass transit system. When these factors, as well as others were added to their logistic regression model, the only significant predictor for a temporary threshold shift after riding was heavy transit use (OR = 2.9), and female gender (OR = 2.7). Overall, more studies characterizing the impact of concurrent use of MP3 players and lengthy transit times, as well as definitive audiometric evaluation of transit users would continue to clarify the relationship between transit noise exposure and hearing health.

Aside from the cross-sectional design, other limitations of our study include the lack of modeling of other potential factors that may contribute to noise exposure for personal transportation modalities, as well as buses, and streetcar. Although we chose the busiest routes for streetcar and bus modalities of transportation, the relative sample size may be relatively low and may not represent the entire sprawling Toronto transit system. Despite these limitations, these findings still illustrate that the potential noise exposure for Toronto commuters add to the risk for the development of NIHL, not to mention the other adverse health effects from excessive noise.

## Conclusion

Given sufficient exposure duration, noise levels associated with mass transit within the system are intense enough to produce NIHL in users. Furthermore, noise exposures from personal transportation modalities in an urban city, particularly cycling are also sufficiently intense to produce NIHL. As the mass transit system in Toronto continues to expand, engineering noise-control efforts should continue to focus on materials and equipment that confer a quieter environment. Hearing protection while using public transit should also be promoted, and further studies characterizing the risk of developing NIHL should be pursued.

## Additional file

Additional file 1: Table S1. Toronto Subway noise measurements by station. (DOCX 20 kb)

## Abbreviations

CDC: the Center for Disease Control and Prevention
dBA: A-weighted decibels
EPA: the US Environmental Protection Agency
HL: Hearing Loss
NIHL: Noise Induced Hearing Loss
NIOSH: US National Institute for Occupational Safety and Health
OSHA: US Occupational Safety and Health Administration
WHO: World Health Organization
