# Supplementary material for: Noise exposure while commuting in Toronto - a study of personal and public transportation in Toronto
Source: J Otolaryngol Head Neck Surg. 2017 Nov 23;46:62. doi: 10.1186/s40463-017-0239-6 (PMC5700687; doi:10.1186/s40463-017-0239-6)
Supplement: Additional file 1: Table S1. — Toronto Subway noise measurements by station. (DOCX 20 kb) [file 40463_2017_239_MOESM1_ESM.docx]

**Table S1. Toronto Subway noise measurements by station**

|  | **Line** | **Grade** | **Year** | **2014 Ridership** (Weekday Avg) | **Platform** | **Average Peak Noise** (dBa) | **Time weighted average noise** (dBa) |
| --- | --- | --- | --- | --- | --- | --- | --- |
| **Finch** | 1 | u | 1974 | 90 910 | C | 103.9 | 74.4 |
| **North York Center** | 1 | u | 1987 | 24 560 | C |  |  |
| **Sheppard Yonge** | 1 | u | 1974 | 121 410 | C | 106.75 | 84.8 |
| **York Mills** | 1 | u | 1943 | 22 600 | C |  |  |
| **Lawrence** | 1 | u | 1943 | 24 590 | C | 105 | 84.5 |
| **Eglington** | 1 | u | 1954 | 77 530 | C | 103.5 | 75.65 |
| **Davisville** | 1 | s | 1954 | 23 040 | s | 102.9 | 80.6 |
| **St. Clair** | 1 | u | 1954 | 34 760 | S | 105 | 78.6 |
| **Summerhill** | 1 | u | 1954 | 5 770 | S | 107.4 | 76.65 |
| **Rosedale** | 1 | s | 1954 | 6 260 | S | 103.5 | 80.7 |
| **Bloor-Yonge** | 1 | u | 1954 | 409 220 | S | 104.26 | 77.48 |
| **Wellesley** | 1 | u | 1954 | 24 480 | S | 104.8 | 77.8 |
| **College** | 1 | u | 1954 | 47 940 | S | 105 | 78.3 |
| **Dundas** | 1 | u | 1954 | 75 780 | S | 105.15 | 72.9 |
| **Queen** | 1 | u | 1954 | 52 300 | S | 112.35 | 80.7 |
| **King** | 1 | u | 1954 | 61 360 | S | 109.55 | 79.65 |
| **Union** | 1 | u | 1954 | 125 220 | S | 106.25 | 76.2 |
| **St. Andrew** | 1 | u | 1963 | 55 700 | C | 107.4 | 78.75 |
| **Osgoode** | 1 | u | 1963 | 24 590 | C | 107.05 | 79.25 |
| **St. Patick** | 1 | u | 1963 | 32 500 | C | 111.35 | 78.1 |
| **Queen's Park** | 1 | u | 1963 | 39 660 | C | 104.25 | 83.2 |
| **Museum** | 1 | u | 1963 | 9 800 | C | 111.8 | 86 |
| **St. George** | 1 | u | 1963 | 276 260 | C | 111.9 | 83.4 |
| **Spadina** | 1 | u | 1978 | 53 230 | S | 109.8 | 85.5 |
| **Dupont** | 1 | u | 1978 | 16 490 | S |  |  |
| **St. Clair West** | 1 | u | 1978 | 29 490 | S | 109.15 | 85.8 |
| **Eglington West** | 1 | s | 1978 | 19 000 | S | 106.45 | 82.9 |
| **Glencairn** | 1 | s | 1978 | 6 520 | C |  |  |
| **Larence West** | 1 | s | 1978 | 21 420 | C | 102.95 | 80.5 |
| **Yorkdale** | 1 | s | 1978 | 34 160 | C |  |  |
| **Wilson** | 1 | s | 1978 | 23 610 | C |  |  |
| **Downsview** | 1 | u | 1996 | 39 900 | C | 102.3 | 80.8 |
| **Kipling** | 2 | s | 1968 | 58 100 | C | 107.9 | 75.8 |
| **Islington** | 2 | u | 1968 | 42 630 | C |  |  |
| **Royal York** | 2 | u | 1968 | 24 010 | S | 112.75 | 84.75 |
| **Old Mill** | 2 | u | 1968 | 6 530 | S | 109.15 | 84 |
| **Jane** | 2 | u | 1968 | 20 090 | S | 110.15 | 82.3 |
| **Runnymede** | 2 | u | 1968 | 21 030 | S | 113.1 | 84 |
| **High Park** | 2 | u | 1968 | 9 750 | S | 111 | 83.45 |
| **Keele** | 2 | s | 1966 | 17 550 | S | 114.65 | 84.7 |
| **Dundas West** | 2 | u | 1966 | 27 770 | S | 112.75 | 84.85 |
| **lansdowne** | 2 | u | 1966 | 17 850 | S |  |  |
| **Dufferin** | 2 | u | 1966 | 29 710 | S | 114.95 | 82.55 |
| **Ossington** | 2 | u | 1966 | 32 290 | S | 110 | 81.6 |
| **Christie** | 2 | u | 1966 | 12 390 | S | 111 | 84.95 |
| **Bathurst** | 2 | u | 1966 | 35 510 | S | 110.45 | 85.8 |
| **Bay** | 2 | u | 1966 | 31 050 | C | 119.9 | 84.3 |
| **Sherbourne** | 2 | u | 1966 | 25 860 | S | 112.65 | 82.45 |
| **Castle Frank** | 2 | u | 1966 | 7 070 | S |  |  |
| **Broadview** | 2 | u | 1966 | 34 880 | S | 108.8 | 86 |
| **Chester** | 2 | u | 1966 | 6 540 | S |  |  |
| **Pape** | 2 | u | 1966 | 26 670 | S | 109.15 | 83.5 |
| **Donlands** | 2 | u | 1966 | 10 750 | S |  |  |
| **Greenwood** | 2 | u | 1966 | 10 660 | S |  |  |
| **Coxwell** | 2 | u | 1966 | 16 980 | S | 108.7 | 84.9 |
| **Woodbine** | 2 | u | 1966 | 13 570 | S | 110.15 | 83.05 |
| **Main Street.** | 2 | u | 1968 | 5 580 | S | 112.7 | 78.2 |
| **Victoria Park** | 2 | s | 1968 | 29 890 | S | 111.15 | 84.3 |
| **Warden** | 2 | s | 1968 | 32 110 | C | 113 | 80.3 |
| **Kennedy** | 2 | u | 1980 | 107 330 | C | 105.9 | 75.35 |
| **Lawrence East** | 3 | s | 1985 | 7 470 | S |  |  |
| **Ellesmere** | 3 | s | 1985 | 1 410 | S |  |  |
| **Midland** | 3 | e | 1985 | 2 460 | S | 103.8 | 76.7 |
| **Scarborough Center** | 3 | e | 1985 | 26 470 | S | 110 | 81.1 |
| **McCowan** | 3 | e | 1985 | 3 930 | S | 105.95 | 72.4 |
| **Bayview** | 4 | u | 2002 | 9 030 | C |  |  |
| **Bessarion** | 4 | u | 2002 | 2 380 | C |  |  |
| **Leslie** | 4 | u | 2002 | 6 460 | C |  |  |
| **Donmills** | 4 | u | 2002 | 32 900 | C | 103.5 | 75.55 |

**u = underground, s = surface, e = elevated; C = center platform design, S = Side platform design**
